# Glycation potentiates neurodegeneration in models of Huntington’s disease

**DOI:** 10.1038/srep36798

**Published:** 2016-11-18

**Authors:** Hugo Vicente Miranda, Marcos António Gomes, Joana Branco-Santos, Carlo Breda, Diana F. Lázaro, Luísa Vaqueiro Lopes, Federico Herrera, Flaviano Giorgini, Tiago Fleming Outeiro

**Affiliations:** 1 CEDOC, Chronic Diseases Research Centre, NOVA Medical School | Faculdade de Ciências Médicas, Universidade NOVA de Lisboa, Campo dos Mártires da Pátria, 130, 1169-056, Lisboa, Portugal; 2 Instituto de Medicina Molecular, Faculdade de Medicina, Universidade de Lisboa, Lisboa, Portugal; 3 Department of Genetics, University of Leicester, Leicester LE1 7RH, United Kingdom; 4 Instituto de Tecnologia Química e Biológica, Universidade Nova de Lisboa, Estação Agronomica Nacional, Av. da República, Oeiras 2780-157, Portugal; 5 Department of Neurodegeneration and Restorative Research, Center for Nanoscale Microscopy and Molecular Physiology of the Brain (CNMPB), University Medical Center Göttingen, Waldweg 33, 37073 Göttingen, Germany; 6 Max Planck Institute for Experimental Medicine, Göttingen, Germany

## Abstract

Protein glycation is an age-dependent posttranslational modification associated with several neurodegenerative disorders, including Alzheimer’s and Parkinson’s diseases. By modifying amino-groups, glycation interferes with folding of proteins, increasing their aggregation potential. Here, we studied the effect of pharmacological and genetic manipulation of glycation on huntingtin (HTT), the causative protein in Huntington’s disease (HD). We observed that glycation increased the aggregation of mutant HTT exon 1 fragments associated with HD (HTT72Q and HTT103Q) in yeast and mammalian cell models. We found that glycation impairs HTT clearance thereby promoting its intracellular accumulation and aggregation. Interestingly, under these conditions autophagy increased and the levels of mutant HTT released to the culture medium decreased. Furthermore, increased glycation enhanced HTT toxicity in human cells and neurodegeneration in fruit flies, impairing eclosion and decreasing life span. Overall, our study provides evidence that glycation modulates HTT exon-1 aggregation and toxicity, and suggests it may constitute a novel target for therapeutic intervention in HD.

Huntington’s disease (HD) is an autosomal-dominant neurodegenerative disorder affecting 5–10 per 100 000 individuals^1^,^2^,^3^. Its clinical features include progressive motor dysfunction, cognitive impairment, and psychiatric disturbance and dementia^4^. HD is caused by a CAG triplet repeat expansion in exon 1 of the *HTT* gene, which encodes a polyglutamine (polyQ) stretch in huntingtin (HTT) protein. The number of CAG repeats varies from 16 to 35 in healthy individuals, while expansions of >35 CAG repeats are found in HD patients^1^. The length of the polyQ tract in the protein modulates HTT aggregation, thereby causing cytotoxicity by mechanisms that are still not fully understood, with medium spiny neurons in the striatum particularly affected^5^.

Glucose is the major energy supply of neurons and is essential for their survival. However, impaired glucose metabolism can also damage neurons and lead to neurodegeneration. For example, diabetic patients who neglect their circulating glucose levels frequently develop severe neuropathy that results in the amputation of limbs^6^,^7^. Glucose metabolism drives the formation of by-products that are highly reactive with free amino-groups of proteins. This non-enzymatic reaction, named glycation, induces the formation of advanced glycation end-products (AGEs) that frequently have deleterious effects on proteins^8^,^9^. For example, glycation has been reported in several neurodegenerative disorders such as Alzheimer’s and Parkinson’s diseases, where it potentiates the aggregation and toxicity of proteins such as amyloid-β (Aβ) and α-synuclein, respectively^8^,^9^.

Methylglyoxal (MGO) is an unavoidable by-product of glycolysis and the most reactive glycation agent. It is mainly produced by the non-enzymatic decomposition of the phosphate group of the triose phosphates (glyceraldeyde 3-phosphate and dihydroxyacetone phosphate)^8^. MGO can also arise from the interconversion between glyceraldeyde 3-phosphate and dihydroxyacetone phosphate by the triose phosphate isomerase (Tpi), where the enediolate intermediate may leak from the active site of Tpi in a paracatalytical reaction^10^. Moreover, decreased Tpi activity results in an accumulation of dihydroxyacetone phosphate and MGO^11^,^12^. MGO is detoxified by the glyoxalase system [glyoxalases I (Glo1) and II (Glo2)] and by aldose reductases^13^. In particular, we previously showed that Glo1 inactivation induces a strong increase in MGO levels in yeast^14^. TPI1 deficiency increases the levels of DHAP^15^ and, consequently, increases the levels of MGO^16^,^17^. Notably, TPI deficiency in humans results in increased levels of MGO^18^. We recently demonstrated that glycation of α-synuclein - a central player in Parkinson’s disease - potentiates its aggregation and toxicity (submitted manuscript). Thus, we hypothesized that glycation might act as a common cellular mechanism modulating pathogenesis in several neurodegenerative diseases. Although HD is a genetic disorder, both genetic and environmental factors have been found to modulate the age of disease onset and severity of HD^19^,^20^,^21^,^22^. Although no direct correlation between glycation and the pathogenesis of HD has been established thus far, the levels of the receptors for AGEs (RAGE) are increased in HD brains^23^ and in mouse models^24^. In addition, we also found that DJ-1 - an enzyme with glyoxalase and deglycase activity - modulates HTT toxicity^25^. Here, we show that glycation potentiates HTT aggregation, impairs protein clearance and increases neuronal loss in various established models of HD.

## Results

### Glycation induces HTT aggregation in yeast cells

**Figure 1:**
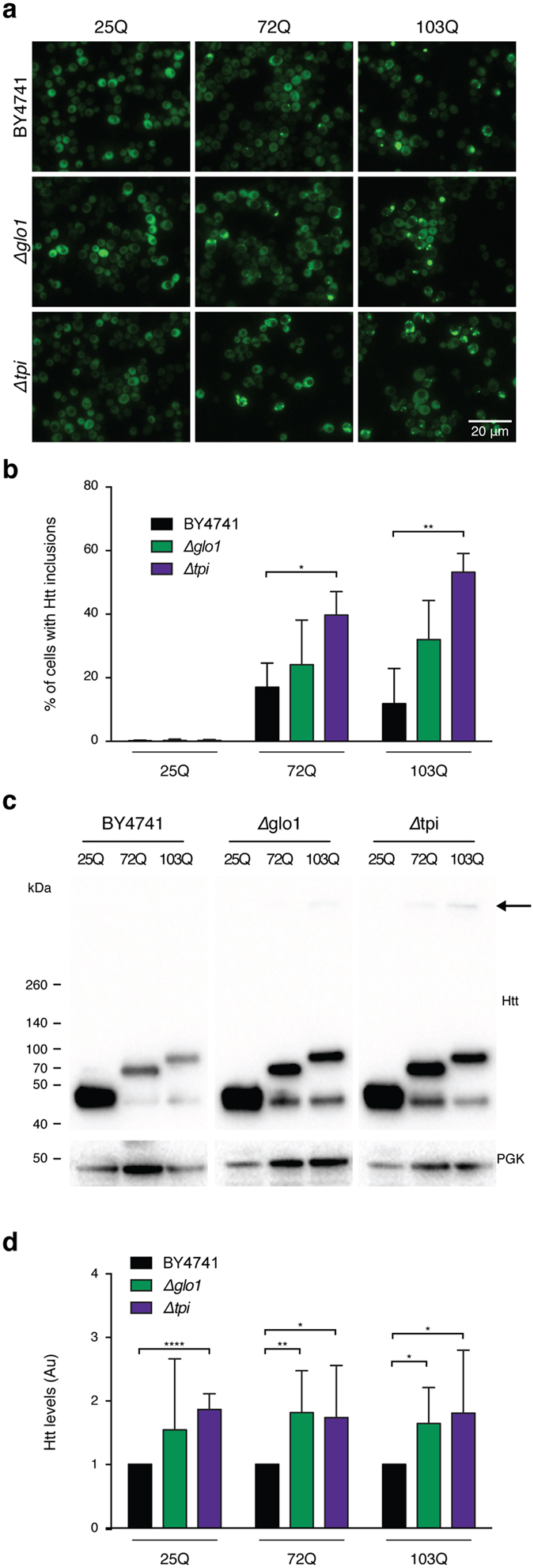
Glycation increases HTT levels and inclusion formation in yeast. **(a)** Fluorescence micrographs of BY4741 (Ctrl), *glo1*Δ and *tpi*Δ yeast strains transformed with HTT 25Q, 72Q or 103Q variants fused to GFP (scale bar 20μm). **(b)** % of yeast cells displaying HTT inclusions (at least n = 3 per condition). **(c)** Yeast protein extracts were immunoblotted with an anti-GFP antibody. Arrow indicates HTT aggregates. **(d)** Corresponding HTT levels are presented in arbitrary units (at least n = 3 per condition). Data in all panels are average ± SD, *p < 0.05, **p < 0.01, ***p < 0.001, ****p < 0.0001. For (*D*), unpaired two-tailed t-test with equal SD.

We started by investigating the effects of MGO in a highly tractable yeast model of HD based on the expression of GFP-tagged HTT exon 1 fragments^26^ with normal (HTT25Q) or expanded (HTT72Q and HTT103Q) polyQ stretches, which lead to HTT aggregation and toxicity^27^,^28^. Interestingly, we observed that glycating conditions (using MGO as the glycating agent) potentiated the formation of HTT72Q and HTT103Q inclusions in a dose-dependent manner, an effect that was potentiated by genetic deletion of Tpi (Fig. 1a,b).

We also modulated MGO levels genetically using *Glo1* (Δ*glo1*) or *Tpi* (Δ*tpi*) knockout strains. Deletion of these genes increases the intracellular levels of MGO and promotes the accumulation of AGEs^14^,^16^,^17^. Remarkably, in protein extracts from Δ*glo1* and Δ*tpi* strains, HTT was retained in the wells of SDS-PAGE gels (Fig. 1c), further demonstrating that glycation promotes aggregation of mutant HTT. HTT aggregation is also a function of HTT intracellular concentration, as overexpression of normal 25QHTT can also lead to aggregation^29^. Consistently, MGO also increased the intracellular HTT levels in a dose-dependent manner (Fig. 1c,d).

### MGO increases HTT levels, aggregation, and toxicity in human cells

**Figure 2:**
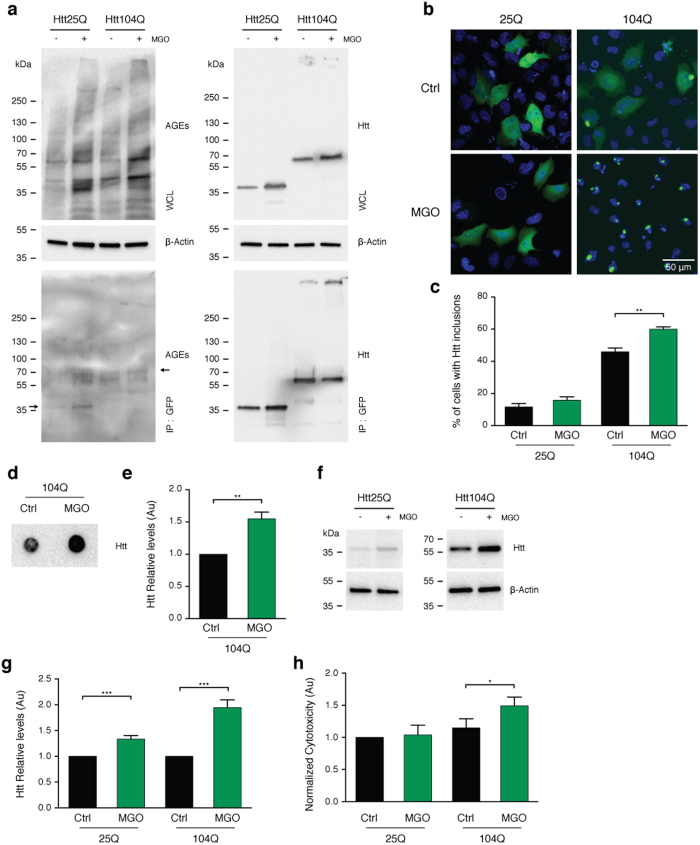
MGO induces HTT aggregation and toxicity in H4 cells. **(a)** Human H4 cells expressing HTT 25Q or 104Q were treated with vehicle (Ctrl) or MGO (0.5 mM) for 16 h. Cells were lysed and immunoprecipitated (IP) with GFP-trap (bottom panels). The whole cell lysates (WCL) and IP samples were probed for AGEs (left panels) or GFP (right panels). Arrow indicates HTT 25Q and HTT 104Q MW. Corresponding loading controls (β-actin) are presented (n = 3). **(b)** H4 cells expressing HTT 25Q or 104Q fused with GFP were treated with vehicle (Ctrl) or MGO (0.5 mM) for 16 h. After treatment, cells were probed with Hoechst and imaged *in vivo*. Fluorescence micrographs and **(c)** corresponding % of cells with HTT inclusions are presented. Scale barm 50 μm. **(d)** Representative filter trap assay and **(e)** corresponding HTT levels of cells expressing HTT 104Q treated with vehicle or MGO for 16 h and immunoblotted with an anti-GFP antibody. **(f)** Protein extracts were immunoblotted with an anti-GFP antibody. **(g)** The corresponding HTT levels are presented (at least n = 3 per condition). **(h)** Toxicity of vehicle (Ctrl) or MGO measured by LDH release (n = 3) and normalized to 25Q. Data in all panels are average ± SD, *p < 0.05, **p < 0.01, ***p < 0.001, ****p < 0.0001. For (**c,e,g,h**), unpaired two-tailed t-test with equal SD.

To further investigate the effects of glycation on HTT, we used transfected human H4 cells with variants of GFP-tagged HTT exon 1 fragments encoding for 25 (HTT25Q) or 104 (HTT104Q) glutamines. To increase protein glycation, cells were treated with 0.5 mM of MGO, a working concentration widely used in various studies^30^,^31^,^32^. We found that this treatment increased protein glycation without increasing overall cytotoxicity (submitted manuscript). We confirmed that MGO treatment increased overall glycation levels of cells expressing either HTT25Q or HTT104Q (Fig. 2a). Moreover, using immunoprecipitation, we detected a consistent increase in the levels of AGEs in both HTT25Q and HTT104Q (Fig. 2a). Treatment with MGO induced a significant increase in the percentage of cells displaying HTT inclusions (1.33 fold) (Fig. 2b,c) and SDS-insoluble aggregates (1.5 fold), as assessed by filter trap assays (Fig. 2d,e). The levels of both HTT25Q and HTT104Q increased significantly under glycating conditions (1.33 and 1.95 fold, respectively) (Fig. 2f,g). Notably, treatment of cells with MGO also specifically increased HTT104Q toxicity (1.5 fold) (Fig. 2h) and decreased cell viability of both HTT25Q and HTT104Q expressing cells (Fig. S1).

### MGO impairs HTT clearance

**Figure 3:**
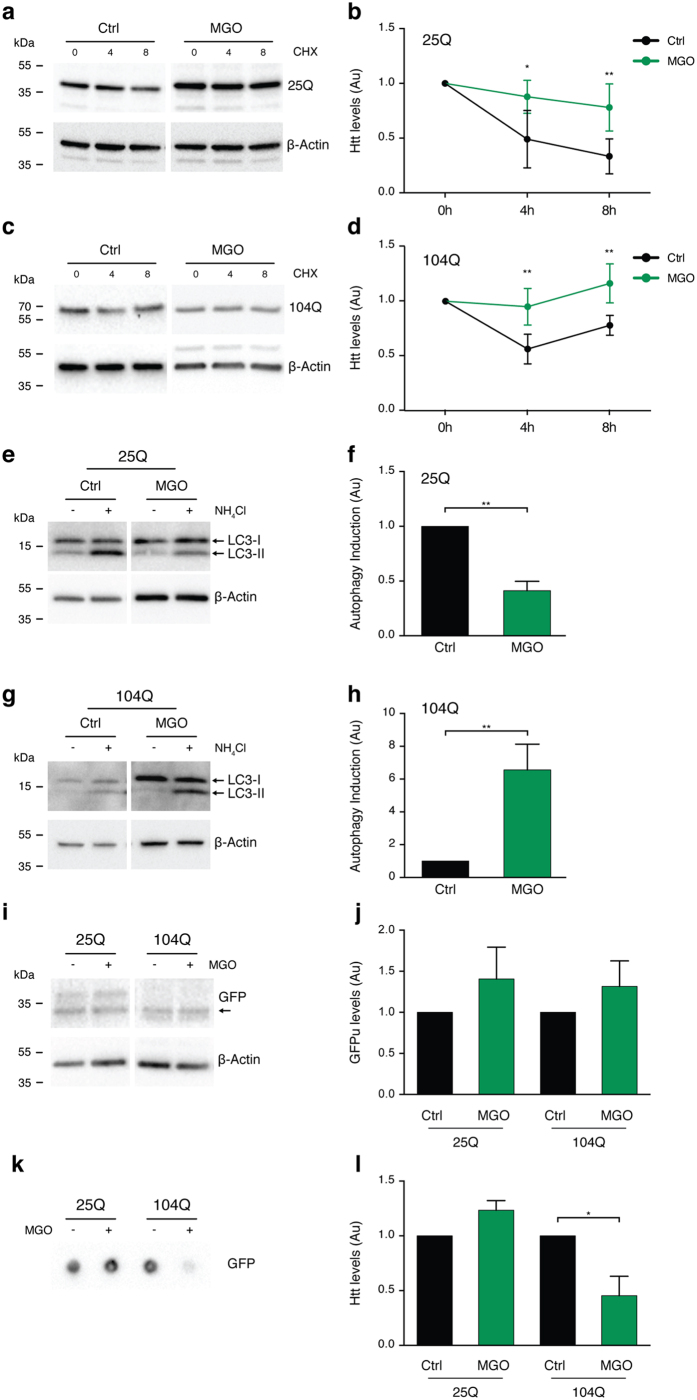
Glycation impairs HTT clearance. H4 cells expressing HTT 25Q (**a,b**) or 104Q **(c,d)** for 24 h were pre-treated with vehicle (Ctrl) or MGO (0.5 mM) for 16 h. Cells were treated with vehicle or MGO for 24 h together with CHX. Protein extracts were probed for GFP and β-actin, for normalization, and protein levels are presented (at least n = 3). HTT 25Q **(e,f)** and 104Q **(g,h)** expressing cells were pre-treated with vehicle (Ctrl) or MGO (0.5 mM) for 16 h. Cells were treated with vehicle or MGO for 2 h together with vehicle (−) or NH_4_Cl (+). Protein extracts were probed for LC3 (I and II) and β-actin. LC3-II levels (lower band) were normalized to β-actin and as a metric for autophagy induction, the difference between NH_4_Cl and vehicle treatments was calculated. The ratio between MGO and Ctrl is presented as autophagy induction ratio (at least n = 3). **(i)** HTT 25Q and GFPu 104Q and GFPu cells were treated with vehicle (−) or MGO (0.5 mM) (+) for 16 h. Protein extracts were probed for GFP and β-actin (at least n = 3). Arrow indicates GFPu. **(j)** Normalized GFPu levels are presented. **(k)** HTT 25Q or 104Q were treated with vehicle (−) or MGO (0.5 mM) (+) for 16h. Fresh media was conditioned for 6 h in the same cells (−) or (+) and probed in a dotblot system for GFP (n = 3). **(l)** Normalized Htt released levels are presented. Data in all panels are average ± SD, *p < 0.05, **p < 0.01. For (**b,d,f,h,j**), unpaired two-tailed t-test with equal SD.

Since MGO increased the intracellular levels of HTT, we next investigated the possible involvement of protein clearance pathways. First, we monitored the rate of clearance of the different HTT variants upon blocking *de novo* protein synthesis using cycloheximide (CHX). Interestingly, clearance of both HTT25Q (Fig. 3a,b) and HTT104Q (Fig. 3c,d) was impaired under MGO-induced glycating conditions.

Second, we assessed the effect of MGO in the autophagy lysosome pathway (ALP). For this, we blocked the ALP system with ammonium chloride (NH_4_Cl) in vehicle or MGO-treated cells, and evaluated the activation of ALP by measuring the accumulation of LC3-II levels^33^. Treatment of cells expressing HTT25Q with MGO reduced the activation of ALP (0.4 fold) (Fig. 3e,f). In contrast, in cells expressing HTT104Q we observed increased activation of ALP (6.6 fold) in response to MGO (Fig. 3g,h).

Third, we evaluated the effects of MGO on the UPS system using an unstable version of GFP (GFPu) that indicates the overall activity of the UPS^34^. Briefly, when the UPS is functioning properly, low levels of GFPu accumulation are expected. As a positive control, we blocked the proteasome function with MG132 and observed a dose-dependent increase in the fluorescence levels, indicating proteasome inhibition (Fig. S2). The levels of GFPu were not altered in cells expressing either HTT25Q or HTT104Q upon MGO treatment (Fig. 3i,j).

Finally, we asked whether glycating conditions affected the cellular release of different HTT exon 1 variants. Conditioned media from HTT104Q-expressing cells treated with MGO showed a significant reduction in the total amount of HTT (less than 0.5 fold), but no changes were observed in the conditioned media from HTT25Q-expressing cells (Fig. 3k,l).

These results suggest that glycation impairs HTT clearance, causing it to accumulate and aggregate within cells.

### Glycation enhances toxicity in *Drosophila* models of HD

**Figure 4:**
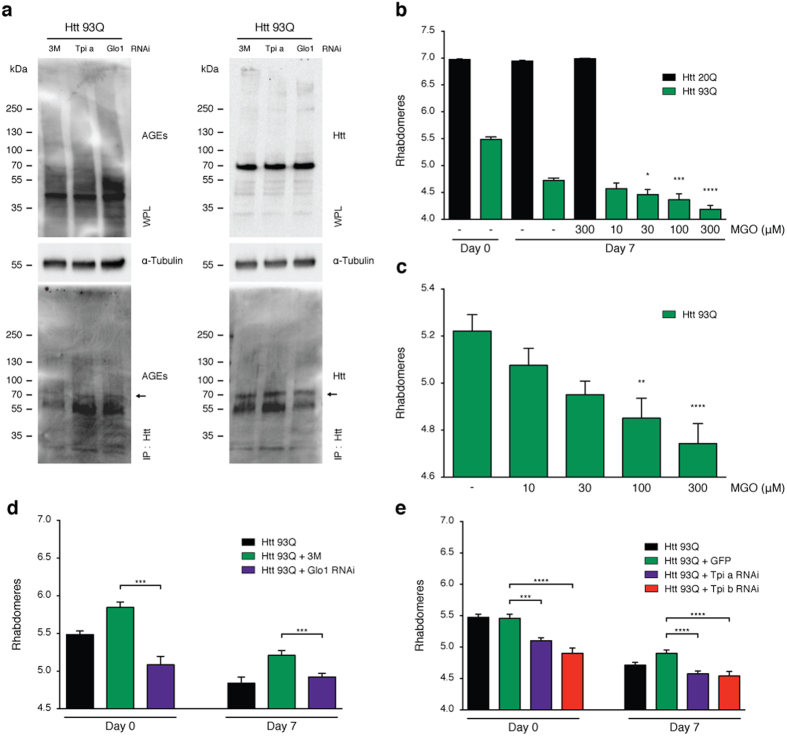
Knockdown of *Glo1* or *Tpi* induces neurotoxicity and decreases lifespan and survival in HTT93Q expressing flies. **(a)** 100 heads of flies expressing Htt93Q and knocked down for *Tpi, Glo1* or 3 M (as control) were lysed and immunoprecipitated with anti-HTT antibody (bottom panels). The whole protein lysates (WPL) (n = 3) and IP samples (n = 2) were probed for AGEs (left panels) or HTT (right panels). Arrow indicates HTT93Q MW. Corresponding loading controls (α-tubulin) are shown. **(b)** Adult flies expressing HTT93Q were treated with different concentrations of MGO. Quantification of mean rhabdomeres per ommatidium is presented. **(c)** Flies were treated during development with MGO. Quantification of rhabdomeres per ommatidium upon eclosion is presented. Number of rhabdomeres per ommatidium in HTT expressing flies with pan-neuronal knockdown of *Glo1* **(d)** or *Tpi* **(e)** is presented at day 0 or 7 post-eclosion. *3M* + *Htt93Q* and *GFP* + *Htt93Q* were used as titration controls for *Glo1* and *Tpi* silencing lines, respectively. Data in all panels are mean ± SEM, *p < 0.05, **p < 0.01, ***p < 0.001, ****p < 0.0001; one-way ANOVA with Newman-Keuls post-hoc test.

**Figure 5:**
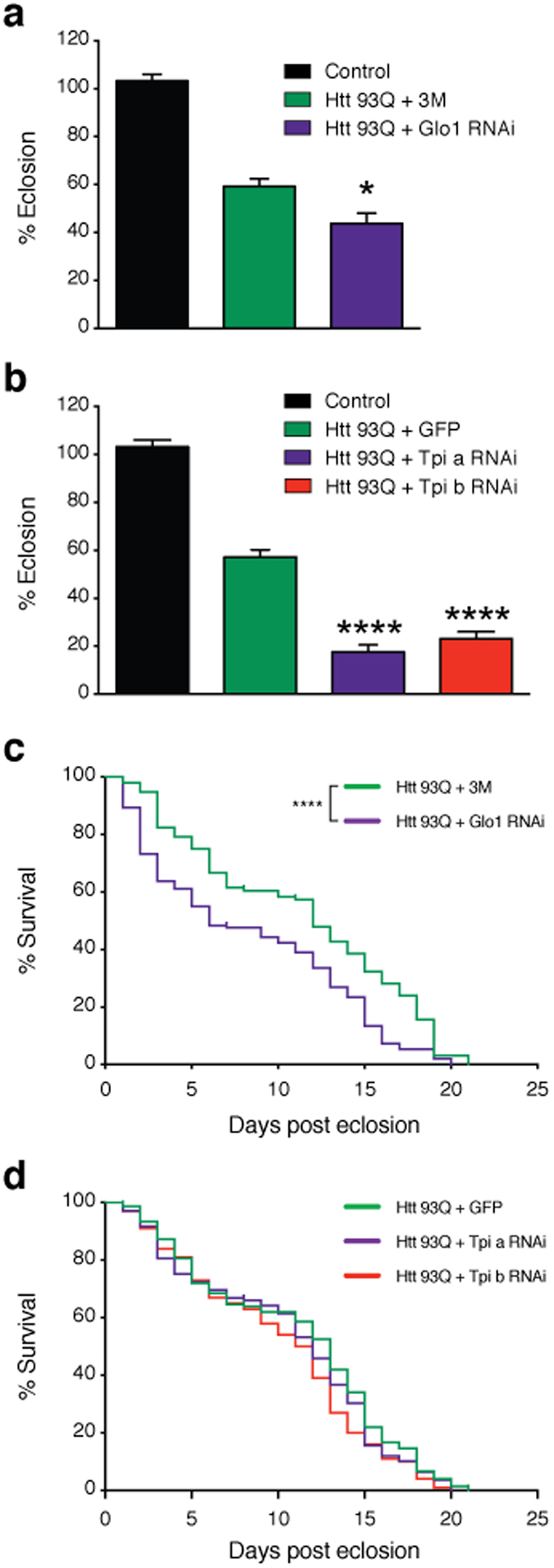
Knockdown of *Glo1* or *Tpi* impairs development and reduces lifespan in flies. RNAi silencing of *Glo1* **(a)** or *Tpi* **(b)** caused a reduction in the percentage of flies emerging from the pupal case. Flies carrying a single copy of the driver (*elavGAL4*) are shown as a control. Data in panels (**a,b**) are mean ± SEM. Survival rate was evaluated in flies with pan-neuronal knockdown of *Glo1* **(c)** or *Tpi* **(d)** in mutant HTT backgrounds (n = 100–150 flies per genotype). *3M* + *Htt93Q* and *GFP* + *Htt93Q* were used in experiments as titration controls for *GloI* and *Tpi* silencing lines, respectively. *Htt93Q* + *Glo1* RNAi (mean = 6); *Htt93Q* + *3M* (mean = 12); *Htt93Q* + *Tpi* a RNAi (mean = 12); *Htt93Q* + *Tpi* b RNAi (mean = 11.5); *Htt93Q* + GFP (mean = 13). *p < 0.05, **p < 0.01, ****p < 0.0001; for (**a,b**) Ordinary one-way ANOVA with Newman-Keuls multiple comparisons test; for (**c**,**d**) Log-rank (Mantel-Cox) test.

Given that MGO increased HTT aggregation and toxicity in cell models, we next investigated whether glycation also affected neuronal loss *in vivo*, employing a *Drosophila* model of HD based upon the pan-neuronal expression of a HTT exon 1 fragment with 93 or 20 glutamines (HTT93Q or HTT20Q) via the GAL4/UAS system^35^. HTT93Q flies exhibit a variety of HD-relevant phenotypes, including decreased lifespan, locomotor defects, degeneration of photoreceptor neurons (rhabdomeres), and impaired emergence of the adult fly from the pupal case (eclosion)^36^,^37^. We manipulated glycation in fruit flies either via MGO administration or genetic knockdown of either *Glo1* or *Tpi*. Importantly, silencing of *Tpi* or *Glo1* in flies expressing HTT93Q displayed an overall increase in glycation and an increase in the levels of AGEs (Fig. 4a). Adult HTT93Q-expressing flies displayed a dose-dependent reduction in the number of rhabdomeres per ommatidium when treated with MGO for 7 days (Fig. 4b). Moreover, we found that treatment of HTT93Q larvae during development with MGO resulted in a significant reduction in the number of rhabdomeres upon eclosion (Fig. 4c). RNAi knockdown of either *Glo1* or *Tpi* in WT flies did not modulate neurodegeneration (Fig. S3). However, in HTT93Q expressing flies, knockdown of either *Glo1* or *Tpi* caused a significant increase in neurodegeneration of rhabdomeres at both 0 and 7 days after eclosion (Fig. 4d,e) when compared to their relative titration controls. Remarkably, we observed a significant reduction in the percentage of eclosion in both *Glo1* and *Tpi* knockdown lines (Fig. 5a,b) and a 6-day reduction in the lifespan in the *Glo1* knockdown line (Fig. 5c,d). These results strongly suggest that glycation can act as an important environmental modifier of HTT toxicity and HD.

## Discussion

The precise mechanisms through which HTT mutations cause HD are still unknown. However, accumulation of HTT plays an important role in the disease and strategies aimed at reducing its levels constitute attractive strategies for therapeutic intervention and are currently being explored in ongoing clinical trials^38^.

Different posttranslational modifications (PTMs) are known to modulate the levels, aggregation propensity and clearance of HTT, and could play a role in the development of HD^39^,^40^,^41^. For example, phosphorylation of HTT on Ser13 and Ser16 is protective in mouse models of HD^42^ enhancing the clearance of HTT via the proteasome and chaperone-mediated autophagy^43^. Acetylation of Lys9 and Lys444 promotes HTT clearance via the autophagy lysosome pathway (ALP)^44^. On the other hand, SUMOylation leads to HTT accumulation and translocation into the nucleus^45^.

Using established yeast, mammalian cell and fly models of HD, we found that both pharmacological and genetic induction of glycation plays a deleterious role in HD models by decreasing HTT clearance, increasing the intracellular levels of HTT and enhancing its aggregation and toxicity, either directly or indirectly (through the increase in the levels of HTT). Impaired clearance of HTT via the proteasome was previously associated with HD^2^. Strikingly, we observed different effects of glycation on normal (25Q) vs. mutant (104Q) HTT variants. While glycation impaired the activation of the ALP system in cells expressing HTT25Q, we observed increased activation of the ALP system in cells expressing HTT104Q. Although we cannot completely exclude an indirect effect of general protein glycation, we clearly observed distinct phenotypes between 25Q and 104Q HTT. The increased ALP activation suggests a compensatory mechanism for the cell to cope with aggregated HTT that is normally cleared via the ALP system^46^,^47^.

Diabetes is a risk factor for neurodegenerative diseases such as Alzheimer’s and Parkinson’s^8^,^9^. A major consequence of diabetes is glucose metabolism imbalance and consequent hyperglycemia. Glucose and its byproducts have the ability to react with amino groups, forming AGEs that can impact the function of target proteins. Glycation exacerbates the accumulation, aggregation and toxicity of Aβ and α-synuclein^8^,^48^. Therefore, it is reasonable to hypothesize that glycation might constitute a common mechanism contributing to the development of neurodegenerative diseases by acting as a “second hit” that tips the proteostasis balance of cells, causing dysfunction of multiple essential cellular pathways, and leading to premature death. Interestingly, and in agreement with our hypothesis, drugs used for the treatment of diabetes were already shown to be protective in HD. For example, metformin was shown to prolong the survival of HD male mice^49^. In addition, exendin-4 decreased HTT aggregation, suppressed cellular pathology in both brain and pancreas, improved motor function, and extended survival in a mouse model of HD^50^. In fact, the prevalence of diabetes is higher in HD patients^51^,^52^. Mouse models of HD develop hyperglycemia^53^,^54^, possibly due to the accumulation of intranuclear HTT inclusions in pancreatic β cells that produce insulin^55^,^56^. Consistently, HD brains differentially express several proteins linked to type-2 diabetes^57^. However, whether diabetes is a contributing factor to pathogenesis or a consequence of HD is still unclear, as is the role of HTT glycation.

Although activation of autophagy could clear aggregated proteins and is considered an important therapeutic approach for several neurodegenerative disorders^58^, autophagy could also lead to neuronal death. Overactivation of ALP can lead to apoptosis or autophagic cell death^59^. Therefore, promoting glycation in HTT104Q expressing cells might account for the observed increase in HTT cytotoxicity by overactivating the ALP.

We did not observe alterations in the UPS, suggesting that, under the conditions tested, glycation does not affect HTT clearance by the proteasome. Interestingly, we observed that glycation reduces the release of HTT104Q, which may account for the observed intracellular accumulation of HTT104Q, increased aggregation and toxicity.

*In vivo*, feeding of mutant HTT exon-1-expressing flies with MGO, reduced the number of rhabdomeres per omatidium, demonstrating a dose-dependent and selective effect. The deleterious effect of MGO was also observed in a reduction of eclosion, suggesting that glycation exacerbates HTT-toxicity during development. Importantly, genetic manipulation of pathways controlling MGO levels, through *Glo1* or *Tpi1* knockdown, also resulted in increased neurotoxicity of mutant HTT93Q, and reduced survival. In *Tpi* RNAi flies, we observed a significant reduction in eclosion, and in *Glo1* RNAi flies we observed a significant reduction in eclosion and survival rate. Altogether, our data clearly suggest that increased glycation exacerbates the toxicity of mutant HTT, and that this can occur already during developmental stages.

**Figure 6:**
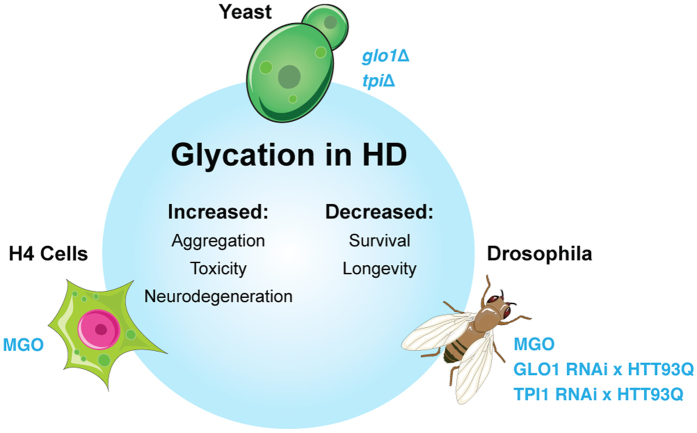
Schematic representation of the effects of MGO glycation in models of HD. Glycation increases HTT intracellular levels and inclusion formation in yeast and H4. Ultimately, it increases HTT-dependent toxicity, leading to neurodegeneration and reduced viability and lifespan in flies.

In this study we demonstrate for the first time that glycation plays an important role in HTT homeostasis, contributing to its neurotoxicity, accumulation and aggregation, as well as potentiating several disease-relevant phenotypes in a *Drosophila* model of HD (Fig. 6). Therefore, we hypothesize that hyperglycemia, and the unavoidable glycation of proteins including HTT, may not only be a consequence but also a contributing factor in HD pathogenesis.

Interestingly, we recently showed that DJ-1 overexpression protects against HTT toxicity in yeast and fruit flies^25^. Although mutations in the DJ-1 gene are associated with recessive forms of Parkinson’s disease, DJ-1 was recently described as an anti-MGO enzyme with glyoxalase activity^60^. Moreover, DJ-1 was also suggested to act as a protein deglycase that repairs MGO-glycated proteins^61^. These findings are consistent with our study, further supporting a connection between protein glycation and HD. Altogether, our study suggests that glycation might contribute to HTT dysfunction and that modulation of glycation may constitute a novel target for therapeutic intervention in HD.

## Materials and Methods

### HTT protein levels and aggregation in yeast

The parental *Saccharomyces cerevisiae* strain BY4741 (*MAT a, his3*Δ, *leu2*Δ, *met15*Δ, *ura3*Δ), and deletion mutants *glo1*Δ and *tpi*Δ strains were used (Euroscarf collection). Cells were grown and transformed as in ref. 13 using GFP fusion constructs with a mutant HTT fragment with an expanded polyQ stretch of either 25, 72 or 103 in p416 vector under the regulation of the GPD promoter as in ref. 26. Protein extraction was performed as in ref. 13. Fluorescence microscopy was performed using a Zeiss Axiovert 200 M.

### MGO purification

MGO was produced as described in ref. 14.

### Immunoprecipitation, microscopy, immunoblotting, toxicity, viability and aggregation assays

H4 neuroglioma cells were maintained, grown and transfected with HTT exon-1 fragments with a polyQ expansions of either 25 or 104 fused to GFP as in ref. 26. Twenty-four hours after HTT25Q or HTT104Q transfection, cells in 35 mm imaging dishes (Ibidi), 60 mm or 100 mm dishes (Corning) were treated with vehicle (PBS) or MGO (0.5 mM) for 16 h. Total protein lysates were obtained as in ref. 13. Htt was immunoprecipitated using GFP-trap (Chromotek) according to the manufacturer instructions. Widefield fluorescent microscope Zeiss Axiovert 200 M (Carl Zeiss MicroImaging) or point scanning confocal microscope Zeiss LSM 710 (Carl Zeiss MicroImaging) were used to visualize HTT inclusions. Immunobloting was performed according to standard procedures as in ref. 13 using the following antibodies: anti GFP (NeuroMab, P42212, 1:3000); anti AGEs (Cosmobio, KAL-KH-001, 1:500); anti LC3 (Nano Tools, 0260-100/LC3-2G6, 1:2000) and anti β-actin (Ambion, AM4302, 1:5000). Cytotoxicity was measured by means of the LDH kit (Clontech), following manufacturer´s instructions. Cell viability was determined using the MTT assay according to standard procedures. Evaluation of HTT aggregation profiles by filter retardation assay was performed as in ref. 62. Briefly, cellulose acetate membranes retain aggregates that are not soluble in SDS (1%) and that are larger than 0.22 μm.

### HTT clearance assay

For CHX chase experiments, H4 cells were transfected as previously^13^ with either HTT25Q or HTT104Q. After 24 h, cells were treated with vehicle (PBS) or MGO (0.5 mM) for 16 h. Media was renewed and cells re-challenged with vehicle or MGO for 24 h in the presence of CHX (100 μM, added at given time points). Protein extracts were immunobloted. Proteasome impairment was assessed as the amount of GFPu accumulation. H4 cells were cotransfected with E.V. with GFPu; HTT25Q with GFPu or HTT104Q with GFPu. 24 h post transfection, media was renewed and cells treated with vehicle (PBS) or MGO (0.5 mM) for 16 h and processed for immunobloting with anti-GFP antibody (NeuroMab, P42212). As positive control, H4 cells were transfected with GFPu and treated with increasing concentrations of MG132 for 24 hours. GFPu average fluorescence level was analyzed by epifluorescence microscopy. For autophagy impairment studies, H4 cells were transfected with HTT25Q or HTT104Q. 24 h post transfection, media was renewed and cells treated for 16 h with vehicle (PBS) or MGO (0.5 mM). Media was again renewed and cells were re-challenged with vehicle or MGO during autophagy blockage with ammonium chloride (20 mM) for 2 h. Autophagy activity was measured as the amount of accumulated LC3-II after treatment with autophagy blockers^33^. For HTT release studies, H4 cells, transfected with HTT25Q or HTT104Q for 24 h were treated with MGO (0.5 mM) for 16 h. Media was renewed, and cells were again treated with MGO (0.5 mM) for 6 h, media collected, applied onto nitrocellulose membranes (0.22 μm, Millipore) using a dot blot apparatus, and immunobloted with anti-GFP by standard procedures.

### Drosophila lines

Flies were raised at 25 °C in LD12:12 on standard maize food. The *elav-GAL4* (c155) and *w; UASeGFP*; + (5431) lines were obtained from the Bloomington Stock Center (Bloomington, Indiana). The *w;* +*; UASHTT93Q* and *w;* +*; UASHTT20Q* transgenic line was kindly provided by J. Lawrence Marsh and Leslie Thompson (University of California, Irvine)^35^. RNAi transgenic lines were obtained from the Vienna *Drosophila* RNAi Center (VDRC). For *Glo1* knockdown, the 101560 line from the KK Library (phiC31-based transgenes at a single, defined site) was used. For *Tpi* knockdown, two lines were employed from the GD Library (P-element based, random insertion sites): 25643 and 25644. The *UASeGFP* and *w; 3M;* + (carrying an empty vector located in the KK site) lines were used in the experiment as titration controls.

### Immunoprecipitation of HTT expressed in flies

Flies were processed for IP 7 days post-eclosion. Briefly, heads were separated using standard procedures, and protein extracts prepared by vigorous homogenization with a tissue grinder followed by liquid nitrogen freeze and thaw cycles. 750 μg of protein were used for IP, as we previously described^63^. 10 μl per IP of anti Htt antibody was used (Millipore, MAB5374). IP and whole protein lysate (WPL) were probed using anti AGEs (Cosmobio, KAL-KH-001, 1:500); anti-Htt (Millipore, MAB5374, 1:1000) and anti α-tubulin (Sigma, T5168, 1:15000).

### Drosophila eclosion and longevity assays

Eclosion of fly lines was assessed as previously described in ref. 64. Male flies carrying the *elavGAL4* driver were crossed to virgin females carrying the UASHTT93Q transgene in order to generate females expressing HTT93Q and control males in the F1 generation. 10–35 crosses were set up in separate vials for each condition and parental flies were removed 5 days after mating. The number of female or male progeny emerging was scored over 10 days post-eclosion and the percentage of eclosion per vial was calculated using the following ratio: (number of female/number of male flies) × 100. For longevity assays, newly emerged female flies of the desired genotype were collected and kept in groups of 10 in separate vials. Flies were moved to fresh food every 2–3 days and the number of dead individuals scored daily.

### Treatment of Drosophila with MGO

MGO was dissolved in H_2_O and added to standard maize media at the required doses (10, 30, 100 or 300 μM). For treatment during development, crosses were set up and flies were grown on standard maize media previously supplemented with MGO. Flies expressing the desired genotype were collected upon eclosion and analyzed for both eclosion percentage and rhabdomere number. For adult feeding experiments, newly emerged flies were transferred to MGO-supplemented food and moved daily to fresh vials with appropriate MGO treatment concentration. Rhabdomeres were scored in flies fed with different concentrations of MGO during 7 days, newly emerged untreated flies and 7 days-old untreated flies.

### Pseudopupil analysis

The number of visible rhabdomeres per ommatidium was measured as described in ref. 37. Briefly, the number of rhabdomeres was scored for a minimum of 50 ommatidia per fly, in at least 12 flies per condition at day 0 or 7 post-eclosion. Heads from adult flies were removed and fixed to glass slides using clear fingernail polish and rhabdomeres were examined at 500X magnification using an Olympus BH2 light microscope.

## Additional Information

**How to cite this article**: Vicente Miranda, H. *et al*. Glycation potentiates neurodegeneration in models of Huntington’s disease. *Sci. Rep.* **6**, 36798; doi: 10.1038/srep36798 (2016).

**Publisher’s note:** Springer Nature remains neutral with regard to jurisdictional claims in published maps and institutional affiliations.

## Supplementary Material

Supplementary Information
