# Supplementary material for: Glycation potentiates neurodegeneration in models of Huntington’s disease
Source: Sci Rep. 2016 Nov 18;6:36798. doi: 10.1038/srep36798 (PMC5114697; doi:10.1038/srep36798)
Supplement: Supplementary Information [file srep36798-s1.pdf]

TITLE

Glycation potentiates neurodegeneration in models of Huntington's disease

AUTHORS

Hugo Vicente Miranda, Marcos António Gomes, Joana Branco-Santos, Carlo Breda, Diana F. Lázaro, Luísa Vaqueiro Lopes, Federico Herrera, Flaviano Giorgini and Tiago Fleming Outeiro*

SUPPLEMENTAL FIGURES

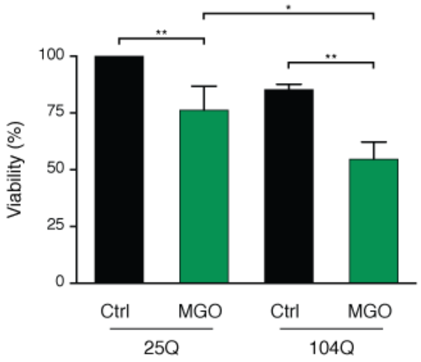

Fig. S1. MGO treatment reduces the viability of H4 cells expressing HTT. H4 cells expressing HTT 25Q or 104Q fused with GFP were treated with vehicle (Ctrl) or MGO (0.5 mM) for 16h. Viability was measured by MTT assay (n=3) and normalized to 25Q. Data in all panels are average ± SD, * $p < 0.05$, ** $p < 0.01$, one-way ANOVA followed by Tukey's multiple comparisons test.

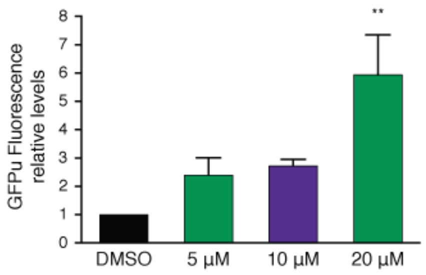

Fig. S2. Proteasome blockade increases GFPu levels. H4 cells expressing GFPu were treated with vehicle (DMSO) or increasing concentrations of MG132 (5-20 μM). Cells were imaged in vivo and the average fluorescence levels normalized to the total number of GFPu expressing cells. Data normalized to DMSO is presented as average ± SD. ** $p < 0.01$, one-way ANOVA followed by Tukey's multiple comparisons test.

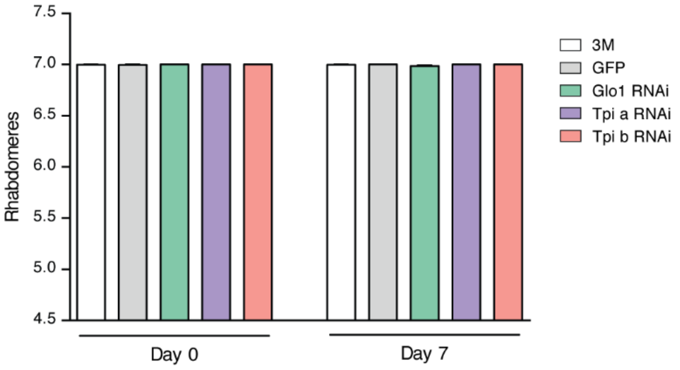

Fig. S3. Knockdown of *Glo1* or *Tpi* does not induce neurotoxicity in WT flies. Number of rhabdomeres per ommatidium in WT flies with pan-neuronal knockdown of *Glo1* or *Tpi* is presented at day 0 or 7 post-eclosion, with no neurodegeneration observed. 3M and GFP expressing control flies also do not exhibit degeneration of rhabdomeres. Data in all panels are mean ± SEM, one-way ANOVA with Newman-Keuls post-hoc test.
